# Phenotypic and genotypic characterization of methicillin-resistant *Staphylococcus aureus* from bovine mastitis

**DOI:** 10.14202/vetworld.2017.363-367

**Published:** 2017-03-30

**Authors:** S. Hamid, Mohd Altaf Bhat, Irfan Ahmad Mir, Anil Taku, Gulzar Ahmad Badroo, Salik Nazki, Andleeb Malik

**Affiliations:** 1 Bacteriology Laboratory, Division of Veterinary Microbiology and Immunology, Sher-e-Kashmir University of Agricultural Sciences and Technology of Jammu, Jammu and Kashmir, India; 2 Laboratory of Veterinary Immunology, College of Veterinary Medicine, Chonbuk National University, Iksan, South Korea

**Keywords:** bovine, mastitis, methicillin-resistant *Staphylococcus aureus*, *mecA*, methicillin-sensitive *Staphylococcus aureus*, *Staphylococcus aureus*

## Abstract

**Aim:**

This study was conducted to determine the occurrence of methicillin-sensitive and *Staphylococcus aureus* (MSSA) methicillin-resistant *S. aureus* (MRSA) from bovine mastitis and to characterize them with respect to antibiotic resistance gene *mecA*.

**Materials and Methods:**

A total of 160 mastitic milk samples were screened for the presence of *S. aureus*. The presumptive positive isolates were confirmed using *nuc* and *23S rRNA* gene-based polymerase chain reaction. All the confirmed isolates were subjected to *in vitro* antibiogram using a number of antibiotics. Isolates which showed resistance against methicillin were characterized for the presence of *mecA* gene.

**Results:**

Out of the total 160 milk samples, 36 (22.5%) samples yielded *S. aureus*. The *in vitro* antibiogram revealed that 16.6% *S. aureus* isolates were resistant to all antibiotics screened for and 5.5% isolates were sensitive to all of them. Furthermore, the study found 94.4%, 83.3%, 77.7%, 66.6%, 50%, and 27.7% of *S. aureus* isolates resistant to penicillin, ampicillin, amoxicillin-sulbactam, enrofloxacin, ceftriaxone, and methicillin, respectively. Out of the 36 *S. aureus* isolates, only 6 (16.6%) isolates were confirmed as MRSA while rest were MSSA.

**Conclusion:**

The higher occurrence of *S. aureus*-mediated mastitis was concluded due to improper hygienic and poor farm management. The multiple drug resistance reveals the indiscriminate use of drugs and presence of methicillin resistance gene determinant is an alarming situation as such infections are difficult to treat.

## Introduction

*Staphylococcus aureus* is a Gram-positive coccal bacterium found commonly as commensal on skin and mucous membrane of upper respiratory and lower urogenital tract of humans and various animal species [1,2]. It has an ability to colonize and infect a variety of host species, including humans, farm and companion animals and wildlife. In case of animals, it is a primary and most lethal agent because it causes chronic and deep infections in the mammary glands that is extremely difficult to be cured [3]. *S. aureus* is the most prevalent and contagious pathogen that is found in 30-40% of all mastitic cases and 80% of subclinical bovine mastitis which may result in about 35 billion US dollar annual losses worldwide [4,5].

The mastitis causing ability is governed by a number of virulence factors which are either structural or secretory [6]. The structural virulence factors include protein A, capsular polysaccharide, peptidoglycan and adhesions, while as secretory includes coagulase, lipase, hyaluronidase, proteases and hemolysins [7,8].

Over the recent past, it has become difficult to control the virulent strains of *S. aureus* from causing mastitis. The reason being some strains of *S. aureus* have become resistant to various β-lactam antibiotics including the powerful ones in this class, i.e., methicillin. Such strains of *S. aureus* are known as methicillin-resistant *S. aureus* (MRSA) [9]. Methicillin resistance is caused by the acquisition of a *mecA* gene. This produces an alternative penicillin-binding protein 2a (PBP2a), which has lower affinity for β-lactam antibiotics [10].

The increase in global prevalence of virulent and multidrug resistant *S. aureus* strains poses a renewed threat to the dairy industry which lays need to undertake research for addressing the issue. Hence, this study was aimed at isolation and molecular characterization of *S. aureus* isolates obtained from mastitic milk of various dairy farms in Jammu for methicillin governing gene determinant.

## Materials and Methods

### Ethical approval

The approval from the Institutional Animal Ethics Committee to carry out this study was not required as no invasive technique was used. The milk samples were collected from clinically affected animals with mastitis as per standard collection procedure.

### Sampling

This study was based on a total of 160 milk samples (80 cows and 80 buffaloes) collected from clinical as well as subclinical mastitis cases. Before sampling, the udders were inspected for externally observed pathological condition. Each milk sample was collected aseptically in a sterile screw-capped bottle from each mammary gland after washing with water and cleaning the teats with cotton soaked in 70% ethanol. The samples were immediately taken to the laboratory for bacteriological analysis.

### Isolation

Isolation of *S. aureus* was attempted according to the method previously described by Singh and Prakash with slight modification [11]. Enrichment was carried out in peptone water enrichment broth (HiMedia Pvt. Ltd., India). A loop full of milk sample was inoculated in sterile enrichment broth and incubated for 24 h at 37°C. Subsequently, a loop full of inoculum from enrichment broth was streaked on Baird Parker agar (HiMedia Pvt. Ltd., India). After 48 h of incubation at 37°C, colonies appeared as jet black surrounded by a white halo characteristic of *S. aureus*. The pure cultures were streaked on Nutrient agar slant and incubated for 24 h at 37°C and then stored at 4°C. The confirmation of the isolates as *S. aureus* was done by demonstration of the typical cellular morphology in Gram stained smears (Gram-positive cocci that occurred in clusters) and biochemical tests such as catalase, oxidase, DNase and coagulase, respectively.

### Extraction of DNA

Suspensions of the bacterial colonies maintained on the nutrient agar slant were prepared in 1.5 ml microcentrifuge tubes in 250 μl of sterile double distilled water by gentle mixing. The samples were boiled for 10 min, cooled on ice for 10 min and centrifuged at 10,000×*g* for 5 min. 2 μl of the supernatant was used as the template for each polymerase chain reaction (PCR).

### Molecular detection of *S. aureus*

The presumptive *S. aureus* isolates were confirmed by amplifying species-specific thermonuclease (*nuc*) gene as described previously [12]. The sequence of primers used is mentioned in Table-1. Isolates negative for *nuc* gene were subjected to another round of PCR using species-specific primers targeting the *23S rRNA* gene [13].

**Table-1:** The sequence of the primers for PCR amplification specific to different genes of *Staphylococcus aureus.*

Primer name	Primer sequence (5’–3’)	Reference	Size (bp)
*nuc*-1 F	GCGATTGATGGTGATACGGTT	Louie *et al*. [12]	270
*nuc*-2 R	AGCCAAGCCTTGACGAACTAAAGC		
Staur 4	ACGGAGTTACAAAGGACGAC	Straub *et al*. [13]	1250
Staur 6	AGCTCAGCCTTAACGAGTAC		
*mecA*F	AAAATCGATGGTAAAGGTTGGC	Louie *et al*. [12]	530
*mecA*R	AGTTCTGCAGTACCGGATTTGC		

PCR=Polymerase chain reaction

### Phenotypic detection of MRSA by oxacillin screening agar test

A bacterial suspension equal to 0.5 McFarland was smeared onto oxacillin resistance screening agar (ORSA) (HiMedia Pvt. Ltd., India), and the plate was incubated at 37°C for 24 h. Oxacillin resistance was confirmed by bacterial growth after 24 h of incubation at 37°C.

### Antimicrobial sensitivity test

MRSA and methicillin sensitive and *S. aureus* (MSSA) isolates were subjected to disc diffusion antimicrobial sensitivity test on Mueller-Hinton agar plates (HiMedia, Mumbai, India) as per the previous protocol described by Bauer *et al*. [14]. The six different antimicrobial discs (HiMedia, Mumbai, India) used in this study were ampicillin, amoxicillin-sulbactam, ceftrioxone, enrofloxacin, methicillin, and pencillin.

### Molecular detection of MRSA targeting mecA gene

All the *S. aureus* isolates were screened for the presence of *mecA* gene as per the method earlier described [12].

## Results

**Figure-1:**
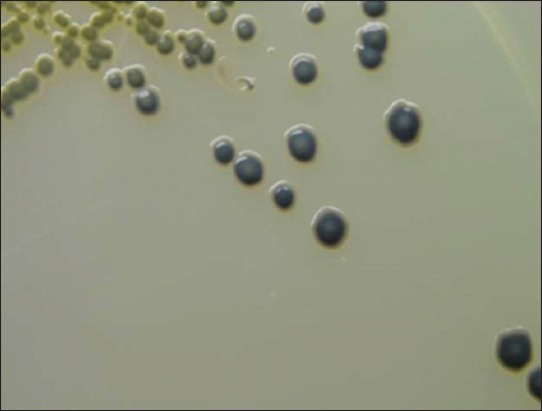
Typical jet back colonies of *Staphylococcus aureus* surrounded by white halo showing lecithinase activity on Baried Parker agar.

**Figure-2:**
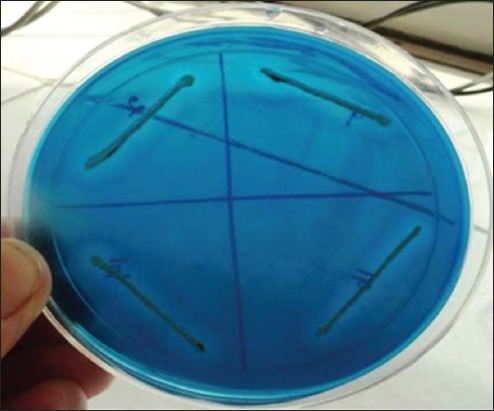
*Staphylococcus aureus* showing DNase activity on DNase agar.

From a total of 160 milk samples collected, 132 (82.5%) samples revealed jet black colonies surrounded by white halo indicating lecithinase activity on Baired Parker agar medium indicating presumptive *S. aureus* as shown in Figure-1. The Gram-staining showed all these isolates were Gram-positive cocci, but only 96 (72.7%) isolates showed characteristic positive catalase and negative oxidase tests and were regarded as presumptive *S. aureus*. From the 96 isolates, 72 (75%) isolates were positive for DNase test and showed typical depolymerization of DNA around the bacterial colonies on DNase agar as shown in Figure-2.

### Molecular detection of *S. aureus*

**Figure-3:**
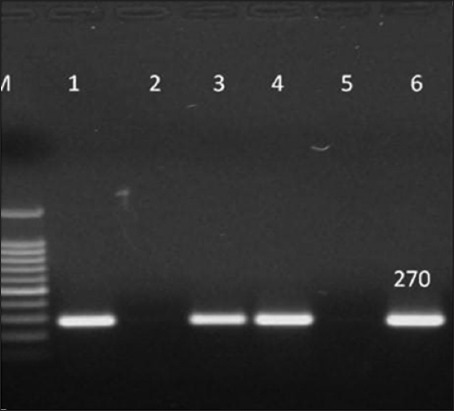
Polymerase chain reaction amplification of species-specific thermonuclease gene (*nuc*) of presumptive *Staphylococcus aureus* isolates. Lane M: 100 bp ladder, Lane 1: Positive control of *S. aureus*, Lane 2: Negative control having distilled water as template, Lane 3, 4, 6: Isolate from mastitic milk samples positive for *S. aureus* indicated by 270 bp band, Lane 5: Isolate from mastitic milk samples negative for *S. aureus*.

**Figure-4:**
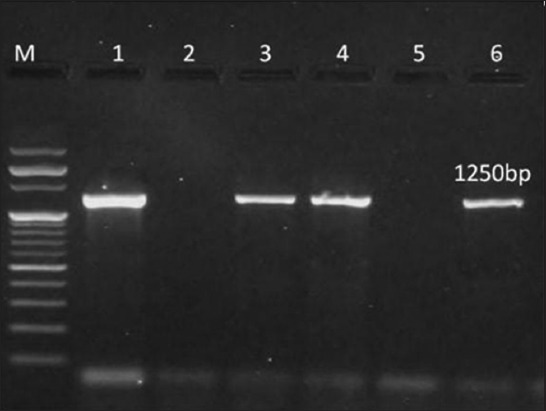
Polymerase chain reaction amplification of species-specific 23S rRNA gene (*staur*) of presumptive *Staphylococcus aureus* isolates. Lane M: 100 bp ladder, Lane 1: Positive control of *S. aureus*, Lane 2: Negative control having distilled water as template, Lane 3, 4, 6: Isolate from mastitic milk samples positive for *S. aureus* indicated by 270 bp band, Lane 5: Isolate from mastitic milk samples negative for *S. aureus*.

Out of the 96 presumptive *S. aureus* isolates, 28 were confirmed as *S. aureus*, as revealed by the amplification of 270 bp product of *nuc* gene specific for *S. aureus* (Figure-3). Out of the *nuc* gene negative isolates, 8 more were confirmed *S. aureus* in species-specific PCR targeting *23S rRNA* gene (Figure-4). Thus, out of 96 presumptive isolates of *S. aureus*, 36 (37.5%) were found positive on the basis of molecular tests.

### Phenotypic detection of MRSA isolates on ORSA

**Figure-5:**
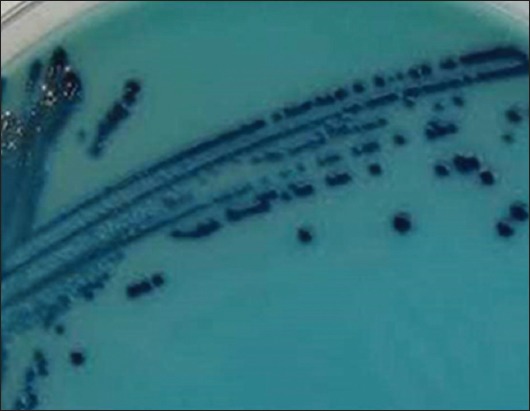
Typical blue colonies of *Staphylococcus aureus* on oxacillin resistance screening agar showing phenotypic methicillin resistant *S. aureus* isolates.

The methicillin resistance was ascertained to check the sensitivity and specificity of MRSA isolates in comparison to molecular detection of MRSA. Only 20 isolates showed growth on ORSA showing lesser sensitivity and specificity than molecular methods (Figure-5).

### Antibiogram of *S. aureus* isolates

Antibiotic sensitivity assay revealed that out of 36 isolates, 34 (94.4%) of isolates were resistant to penicillin, 30 (83.3%) to ampicillin, 28 (77.7%) to amoxicillin-sulbactam, 24 (66.6%) to enrofloxacin, 18 (50%) were resistant to ceftriaxone, and 10 (27.70%) were resistant to methicillin. Two isolates were sensitive to all antibiotics used while as six isolates turned out to be resistant to all of them. The 10 methicillin-resistant isolates on antibiogram included six MRSA isolates which were confirmed by the presence of *mecA* gene.

### Molecular detection of MRSA targeting *mecA* gene

**Figure-6:**
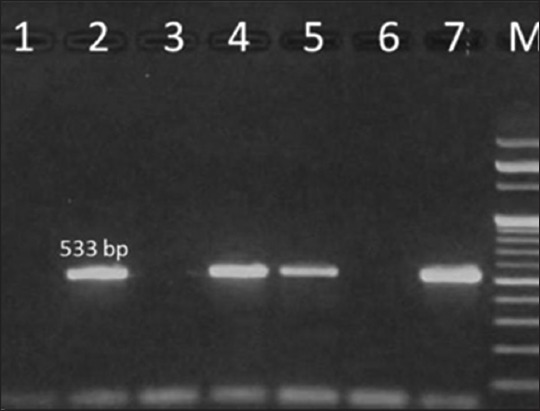
Polymerase chain reaction (PCR) amplification of *mecA* gene of *Staphylococcus aureus* isolates. Lane M: 100 bp ladder, Lane 1: Negative control having distilled water as template, Lane 2: Positive control of *S. aureus* showing PCR amplified product of 533 bp specific for *mecA* gene, Lane 4, 5, 7: *S. aureus* isolate from milk having *mecA* gene, Lane 3 and 6: *S. aureus* isolate from mastitic milk samples.

Out of the total 36 confirmed *S. aureus* isolates, 6 (16.6%) isolates were confirmed to be MRSA when subjected to PCR amplification using specific primers for *mecA* gene. The PCR product appeared as a single band with a size close to the expected size of 533 bp (Figure-6).

## Discussion

*S. aureus* causes one of the most common types of chronic mastitis in dairy animals worldwide. It has been found responsible for more than 80% of subclinical bovine mastitis which results in huge economic losses to farmers in India [15]. In our study, out of 160 mastitic milk samples collected from different regions of Jammu province of India, only 22.5% of cases were found due to *S. aureus*. These results are comparatively lower than those formerly documented by some authors [16,17] who observed a prevalence of 30.6% and 55% which may be possibly because of improper hygiene and poor farm management practices. However, Unnerstad *et al*. [18] have also reported that the most frequently isolated bacterial species was *S. aureus* constituting 21.3% of the diagnoses carried out national wide in Sweden. *S. aureus* isolates can be easily identified by cultural and biochemical tests, but these phenotypic characterization procedures are relative, so it is common practice to confirm the isolates with molecular tests. Two molecular tests one targeting the thermonuclease (*nuc)* gene [12] and other targeting the *23S rRNA* gene [13] are well established. Only 28 (29.1%) isolates could be confirmed by *nuc* gene. However, *nuc* gene negative isolates showed further eight isolates positive for *S. aureus* in *23S rRNA* specific-PCR. Hence, it is recommended that for molecular identification of *S. aureus* both *nuc* and *23Sr RNA* gene-specific PCR should be carried out. Even, when both the genes were targeted only 36 (37.5%) of presumptive isolates could be confirmed. This detection level of *S. aureus* from mastitic milk is low when compared with that of Kuzma *et al*. [19] and Bharathy *et al*. [20] who detected 100% and 65.57% of isolates positive by PCR. The probable reason may be due to other etiological factors belonging to Enterobacteriaceae associated with mastitis.

MRSA is a feature of modern day health-care across the world. Methicillin resistance in staphylococci is mediated by the *mecA* gene, encoding the PBP2a, which has a reduced affinity for the penicillinase resistant penicillins like methicillin and oxacillin and for all other beta-lactam antibiotics [21]. In this investigation, 55.5% of the isolated *S. aureus* were phenotypically resistant to oxacillin and only 16.6% of isolated *S. aureus* strains were carrying the *mecA* gene and identified as MRSA. The variation in the phenotypic and the genotypic results may be due to the presence of MRSA encoding a divergent *mecA* gene. This divergent homolog is referred as *mecC*.

The emergence of antimicrobial resistance among animal pathogens is of growing concern in veterinary medicine as it renders antimicrobials ineffective for treating the animals. The antibiogram pattern revealed that 94.4% of all the isolates were resistant to penicillin. Similar results were obtained by Sori *et al*. [22] who reported that out of 86 pure isolates of *S. aureus* 87.2% were resistant to penicillin while as Khakpoor *et al*. [23] in other study reported that all isolate of *S. aureus* from mastitic cattle were resistant to penicillin. The occurrence of 83.3% resistance to ampicillin in this study is much higher than that of 41.44% as reported by Mubarack *et al*. [24] and lower as compared to 100% resistance reported by Khakpoor *et al*. [23]. In this study, 66.6% isolates turned out to be resistant to enrofloxacin. This is quite contrary to Hussain *et al*. [25] who report 94.73% and 69.56% *S. aureus* isolates were susceptible to enrofloxacin, respectively. The 50% resistance to ceftriaxone is almost in agreement with that of Khakpoor *et al*. [23] who reported 42.10% susceptibility to ceftriaxone.

## Conclusion

Improper hygiene and poor farm managemental practices in the dairy farms could be responsible for contamination of udder resulting in mastitis. There is higher occurrence of *S. aureus*-mediated mastitis in Jammu region. For molecular identification of *S. aureus* both thermonuclease (*nuc)* gene and *23S rRNA* gene-specific PCR should be used simultaneously as none of these tests cover all the *S. aureus* isolates individually. The variation in the phenotypic and the genotypic typing of *S. aureus* as MRSA and MSSA may be due to the presence of MRSA encoding a divergent *mecA* gene like *mecC*. Hence, it is recommended that both the variants should be covered for genotypic typing of *S. aureus*. Unusually, high resistance of *S. aureus* isolates to enrofloxacin indicates its indiscriminate use in clinical practice, hence alarming.

## Authors’ Contributions

AT and MAB designed the study. Laboratory work was done by SH and AM. IAM and GAB prepared the manuscript and analyzed the data. SN done sampling of animals and isolation. All authors read and approved the final manuscript.

## References

[1] Johnson R.C, Ellis M.W, Schlett C.D, Millar E.V, LaBreck P.T, Mor D, Elassal E.M, Lanier J.B, Redden C.L, Cui T, Teneza-Mora N, Bishop D.K, Hall E.R, Bishop-Lilly K.A, Merrell D.S (2016). Bacterial etiology and risk factors associated with cellulitis and purulent skin abscesses in military trainees. PLoS One.

[2] Vanderhaeghen W, Cerpentier T, Adriaensen C, Vicca J, Hermans K, Butaye P (2010). Methicillin-resistant *Staphylococcus aureus* (MRSA) ST398 associated with clinical and subclinical mastitis in Belgian cows. Vet. Microbiol.

[3] Artursson K, Söderlund R, Liu L, Monecke S, Schelin J (2016). Genotyping of *Staphylococcus aureus* in bovine mastitis and correlation to phenotypic characteristics. Vet. Microbiol.

[4] Reshi A.A, Husain I, Bhat S.A, Rehman M.U, Razak R, Bilal S, Mir M.R (2015). Bovine mastitis as an evolving disease and its impact on the dairy industry. Int. J. Curr. Res. Rev.

[5] Wells S.J, Ott S.L, Hillberg-Seitzinger A (1998). Key health issues for dairy cattle-new and old. Symposium emerging health issues. J. Dairy Sci.

[6] Quinn P.J, Markey B.K, Leonard F.C, Hartigan P, Fanning S, Fitz-Patrick E.S (2011). Veterinary Microbiology and Microbial Disease.

[7] Fagundes H, Oliveira C.A.F (2004). *Staphylococcus aureus* intramammary infections and its implications in public health. Cien. Rural.

[8] Iqbal Z, Seleem M.N, Hussain H.I, Huang L, Hao H, Yuan Z (2016). Comparative virulence studies and transcriptome analysis of *Staphylococcus aureus* strains isolated from animals. Sci. Rep.

[9] Robinson D.A, Enright M.C (2003). Evolutionary models of the emergence of methicillin resistant *Staphylococcus aureus*. Antimicrob. Agents Chemother.

[10] Rajabiani A, Kamrani F, Boroumand M.A, Saffar H (2014). Mec-A-mediated resistance in *Staphylococcus aureus* in a referral hospital, Tehran, Iran. Jundishapur J. Microbiol.

[11] Singh P, Prakash A (2008). Isolation of *Escherichia coli*, *Staphylococcus aureus* and *Listeria monocytogenes* from milk products sold under market conditions at Agra region. Acta Agric. Slov.

[12] Louie L, Goodfellow J, Mathieu P, Glatt A, Louie M, Simor A.E (2002). Rapid detection of methicillin-resistant staphylococci from blood culture bottles by using a multiplex PCR assay. J. Clin. Microbiol.

[13] Straub J.A, Hertel C, Hammes W.P.A (1999). 23S rRNA-targeted polymerase chain reaction based system for detection of *Staphylococcus aureus* in meat starter cultures and dairy products. J. Food. Prot.

[14] Bauer A.W, Kirby W.M.M, Sheris J, Truck M (1966). Antibiotic susceptibility testing by a standardized single disk method. Am. J. Clin. Pathol.

[15] Padhy A, Sahu A.R, Shekhar S, Sahoo S, Sahoo A, Dalai N (2015). *Staphylococcus aureus*: An emergent cause of bovine mastitis in India - A review. Int. J. Livest. Res.

[16] Shitandi A, Sternesjo A (2004). Prevalence of multidrug resistant *S. aureus* in milk from large and small scale producers in Kenya. J. Dairy Sci.

[17] Cortimiglia C, Luini M, Bianchini V, Marzagalli L, Vezzoli F, Avisani D, Bertoletti M, Ianzano A, Franco A, Battisti A (2016). Prevalence of *Staphylococcus aureus* and of methicillin-resistant *S. aureus* clonal complexes in bulk tank milk from dairy cattle herds in Lombardy region (Northern Italy). Epidemiol. Infect.

[18] Unnerstad E.H, Lindberg A, Waller P.K, Ekman T, Artursson K, Nilsson-Ost M, Bengtsson B (2009). Carried a nation-wide study on the microbial aetiology of cases of acute clinical mastitis. Vet. Microbiol.

[19] Kuzma K, Malinowski E, Lassa H, Kłossowska A (2003). Specific detection of *Staphylococcus aureus* by PCR in intramammary infection. Bull. Vet. Inst. Pulawy.

[20] Bharathy S, Gunaseelan L, Porteen K, Bojiraj M (2015). Prevalence of *Staphylococcus aureus* in raw milk. Int. J. Adv. Res.

[21] Lozano C, Gharsa H, Ben Slama K, Zarazaga M, Torres C (2016). *Staphylococcus aureus* in animals and food: Methicillin resistance, prevalence and population structure. A review in the African continent. Microorganisms.

[22] Sori T, Hussien J, Bitew M (2011). Prevalence and susceptibility assay of *Staphylococcus aureus* isolated from bovine mastitis in dairy farms of Jimma town, South West Ethiopia. J. Anim. Vet. Adv.

[23] Khakpoor M, Safarmashaei S, Jafary R (2011). Study of milk extracted from cows related to *Staphylococcus aureus* by culturing and PCR. Glob. Vet.

[24] Mubarack H.M, Doss A, Vijayasanthi M, Venkataswamy R (2012). Antimicrobial drug susceptibility of *Staphylococcus aureus* from subclinical bovine mastitis in Coimbatore, Tamil Nadu, South India. Vet. World.

[25] Hussain A, Shakoor A, Yousaf A, Rehman S.U, Zaman M.A (2012). Clinical and subclinical Staphylococcus auerus in dairy buffaloes: Disease characteristics and antibiotic susceptibility profiles of isolates. J. Anim. Plant Sci.

